# siRNA-Mediated suppression of collagen type iv alpha 2 (COL4A2) mRNA inhibits triple-negative breast cancer cell proliferation and migration

**DOI:** 10.18632/oncotarget.13716

**Published:** 2016-11-30

**Authors:** He JingSong, Guan Hong, Jianbo Yang, Zheng Duo, Fu Li, Chen WeiCai, Luo XueYing, Mao YouSheng, OuYang YiWen, Pan Yue, Chang Zou

**Affiliations:** ^1^ Department of Breast Surgery, The First Affiliated Hospital of Shenzhen University, Second People's Hospital of Shen Zhen, Shen Zhen, 518035, China; ^2^ Department of Pathology, The First Affiliated Hospital of Shenzhen University, Second People's Hospital of Shen Zhen, Shen Zhen 518035, China; ^3^ Department of Laboratory Medicine and Pathology, Masonic Cancer Center, University of Minnesota, UMN Twin Cities, MN 55455, USA; ^4^ Shenzhen Key Laboratory of Translational Medicine of Tumor, Department of Cell Biology and Genetics, School of Medicine, Shenzhen University, Shenzhen, Guangdong, 518060, China; ^5^ Department of Pharmacology, Shenzhen Key Laboratory of Translational Medicine of Tumor and Cancer Research Centre, School of Medicine, Shenzhen University, Shenzhen, 518060 China; ^6^ Clinical Medical Research Center, Shen Zhen People's Hospital, The Second Clinical Medical College of Jinan University, 518020, China

**Keywords:** triple-negative breast cancer (TNBC), COL4A2, small interfering RNA (siRNA), lentiviral vector

## Abstract

Triple-negative breast cancer (TNBC) is more aggressive than other breast cancer subtypes. Collagen type IV alpha 2 (COL4A2), a major component of the basement membrane, dynamically influences a wide range of biological processes, including cancer pathogenesis and progression. This study evaluated the effects of COL4A2 siRNA delivered by lentiviral vector to TNBC cells. COL4A2 siRNA lenti-viral vector was constructed and transfected into MDA-MB-231 and MDA-MB-468 cells. The COL4A2 mRNA levels were quantified by RT-PCR. CCK8 assay was performed to evaluate cell proliferation and migration. Cell migration and invasion assays were carried out using Transwell. Cell apoptosis and cell cycle analyses were conducted using flow cytometric approach. We found that COL4A2 mRNA levels were significantly down-regulated in MDA-MB-231 and MDA-MB-468 cells after transfection with COL4A2 siRNA. Furthermore, cell migration and proliferation were significantly decreased and the cell cycle was arrested. Our results indicated that COL4A2 siRNA significantly suppresses the migration and proliferation of TNBC cells. Inhibition of COL4A2 may be a new target for the prevention and treatment of TNBC.

## INTRODUCTION

Breast cancer consists of four molecular subtypes, including luminal A, luminal B, human epidermal growth factor receptor 2 (HER2)-enriched, and triple-negative breast cancer (TNBC) [1–3]. TNBC, which is defined by the negative expression of the estrogen receptor (ER), progesterone receptor (PR) and HER2 receptor, accounts for approximately 15% of all breast cancers [4]. Previous studies suggest that TNBC is more aggressive than other breast cancer subtypes due to a high probability of recurrence and metastasis, causing poor prognosis in patients [5–8]. Although major advances have been achieved in the treatment of ER-positive or HER2-amplified breast cancers, there are no clinically targeted therapies for TNBC, thus leaving cytotoxic chemotherapy as the only choice for systemic therapy [8].

Collagen type IV alpha 2 (COL4A2, MIM 120090), is the major structural component of basement membranes. The C-terminal portion of the protein, known as canstatin, is an inhibitor of angiogenesis and tumor growth [9]. COL4A2 forms heterotrimers together with COL4A1 (MIM120130), which is critical for the stability and functions of vascular basement membrane [10]. In the extracellular space, this kind of heterotrimers polymerize to form flexible sheets, that not only provide structural integrity but also participate in dynamic biological processes, including cell–matrix and cell–cell communication through interactions with growth factors and cell surface receptors [11–14]. Mutations in COL4A2 may contribute to intracerebral hemorrhage (ICH) [15], porencephaly and small-vessel disease with reduced penetrance and variable phenotype [16, 17]. Recently studies revealed that COL4A2 is involved in the pathogenesis and progression of esophageal cancer, lung cancer, and prostate cancer and other cancers.

Previously, it has been shown that COL4A2 is a diagnostic marker signature for esophageal cancer [18]. In addition, COL4A2 is significantly overexpressed in tissues obtained from brain metastases of lung cancer and melanoma patients [19]. Oktem G found a high level of COL4A2 when cancer stem cells (CSC) were maintained as serum-grown prostate CSC spheroids [20]. Furthermore, several studies suggested that COL4A2 may play a role in the pathogenesis of prostate cancer [21], epithelial ovarian cancer [22], colorectal cancer [23], and uterine leiomyoma [24]. A recent study performed in the breast cancer cell line MCF-7 revealed that the reduction of COL4A2 mRNA levels by miR-29b may contribute to the invasiveness of MCF-7 cells [25]. It has been postulated that extracellular matrix-related COL4A2 is associated with breast cancer and may be a potential biomarker for early diagnosis and therapy of breast cancer [26]. All of the aforementioned studies suggest that COL4A2 may play a role in the pathogenesis of cancer, notably breast cancer. However, it remains to be determined if COL4A2 is involved in the cell proliferation and migration of TNBC.

Previously, we reported that COL4A2 was significantly up-regulated in TNBC [27], suggesting that COL4A2 may play an important roles in the pathogenesis of TNBC. In the current study, we used siRNA to suppress COL4A2 expression and investigated how it affects the TNBC cell proliferation and migration.

## RESULTS

### Interference efficiency of siRNA on the expression of Col4A2 mRNA and protein

**Figure 1:**
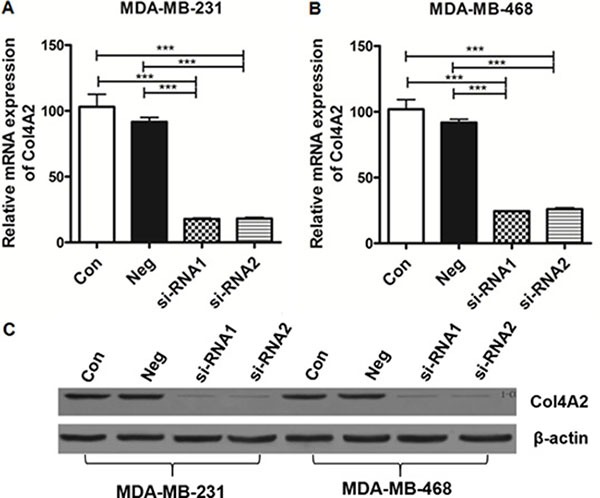
A and B, The relative expression of Col4A2 mRNA and protein in TNBC cells (**A**) MDA-MB-231; (**B**) MDA-MB-468; (**C**) Western blotting validated the interference efficiency.***p < 0.0001 (Con = non-transfected cells;Neg = cells transfected with control shRNA lentiviral vector).

RNA interference was performed in both MDA-MB-231 and MDA-MB-468 cells. As shown in Figure 1A and 1B, RT-PCR results showed the levels of Col4A2 mRNA were significantly lower in the siRNA transfected cells compared to the negative control (NC) (*p* < 0.0001) and control groups (*p* < 0.0001). In addition, the interference efficiency was 82.1% and 74.9% in MDA-MB-231 and MDA-MB-468, respectively.

Western blotting was used to further validate the interference efficiency. Figure 1C showed that the levels of Col4A2 protein were significantly lower in the siRNA transfected cells compared to NC (*p* < 0.0001) and control groups (*p* < 0.0001), and the interference efficiency was 91.3% and 89.4% in MDA-MB-231 and MDA-MB-468, respectively. These results indicated that lentivirus siRNA significantly downregulates the expression of Col4A2 mRNA and protein in TNBC cells.

### siRNA inhibited the proliferation of MDA-MB-231 and MDA-MB-468 cells

**Figure 2:**
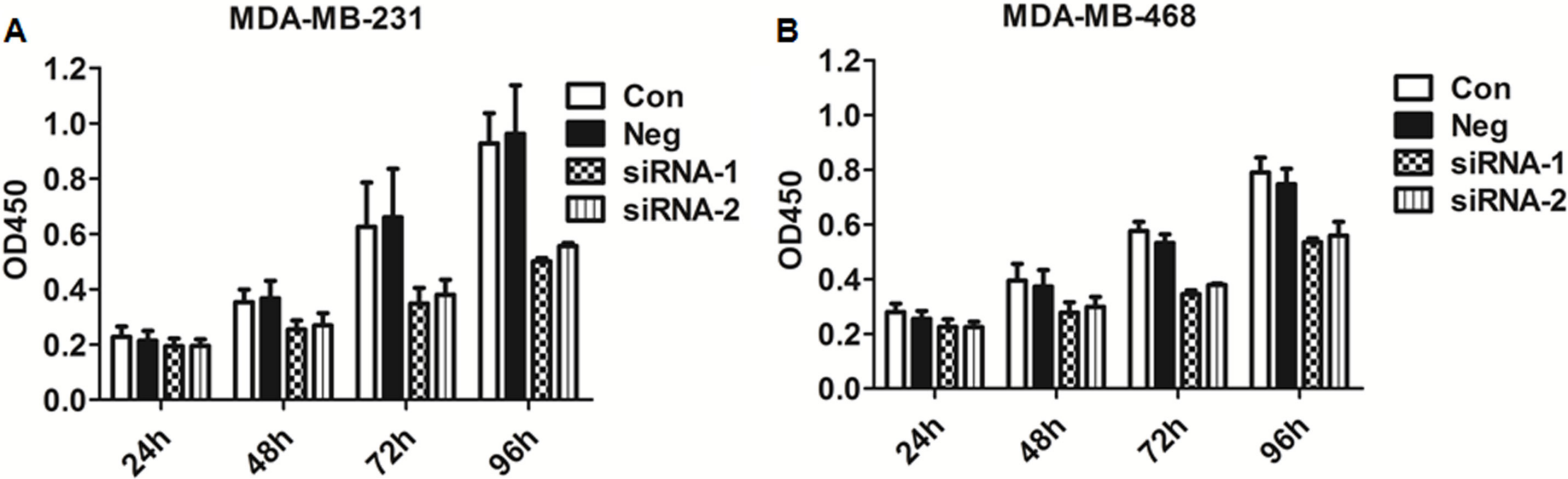
CCK8 assay on cell proliferation at 24, 48, 72, and 96 hours after transfection (**A)** MDA-MB-231; (**B)** MDA-MB-468. (Con = non-transfected cells; Neg = cells transfected with control shRNA lentiviral vector).

The effect of siRNA on cell proliferation was evaluated by CCK8 assay. The results obtained from the CCK8 assay indicated that MDA-MB-231 and MSA-MB-468 cell proliferation was significantly decreased in the siRNA groups 72 h and 96 h after transfection, while there were no significant differences between the NC and control group after 24 h and 48 h after transfection (Figure 2). Together, the above results suggested that Col4A2 lentivirus siRNA significantly inhibits the proliferation of TNBC cells.

### The effect of siRNA on cell migration of MDA-MB-231 and MDA-MB-468 cells

**Figure 3:**
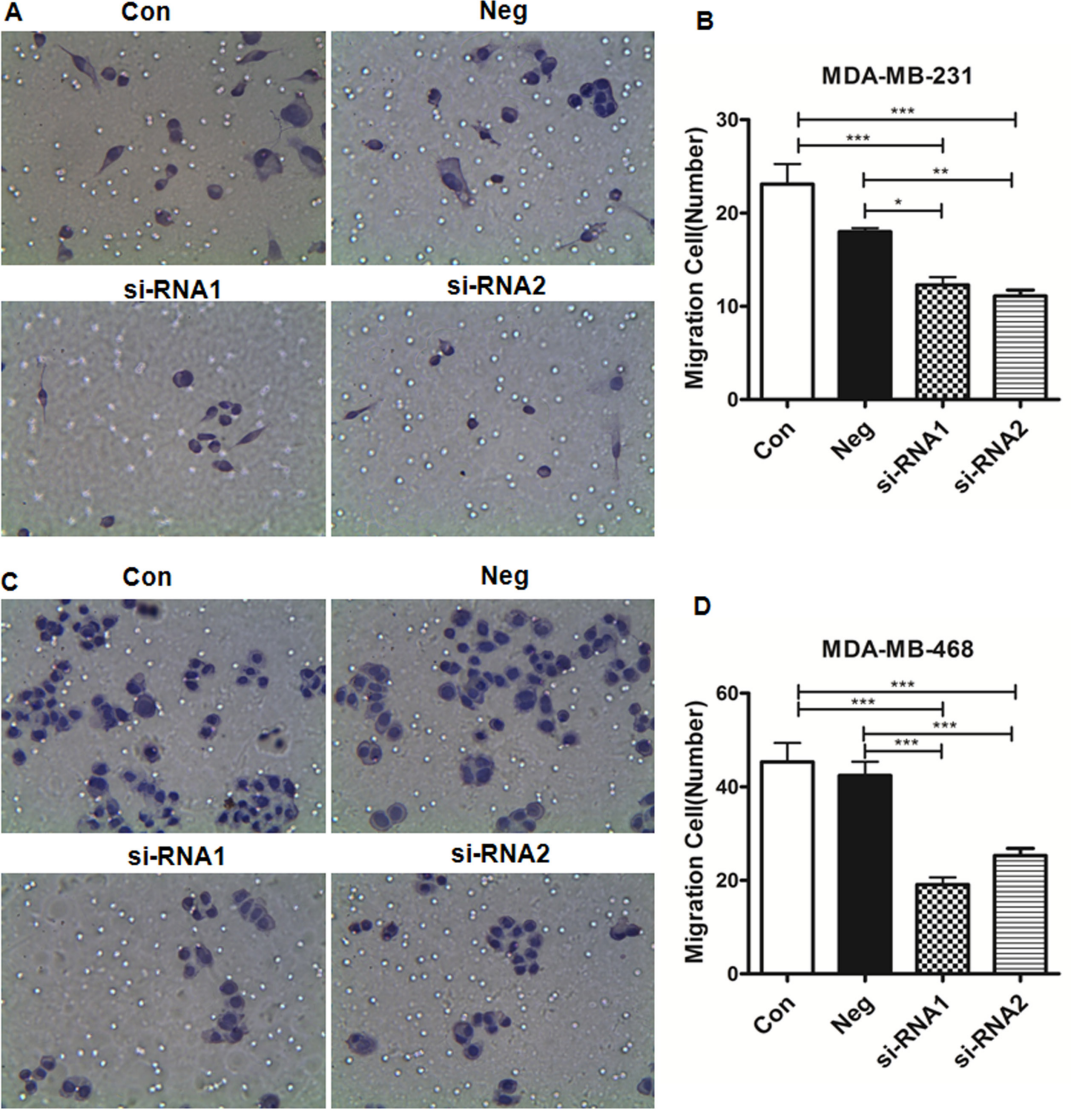
Migration of TNBC cells Light microscopic examination shows the migration of MDA-MB-231 (**A**) and MDA-MB-468 (**C**) cells; the relative migration rate of MDA-MB-231 (**B**) and MDA-MB-468 (**D**). Cells that migrated through the membrane were counted in five random fields for each group, and the relative migration rate= number of migrated cells/number of migrated cells in the control group).*P < 0.05; **P < 0.001; ***P < 0.0001. (Con = non-transfected cells; Neg = cells transfected with control shRNA lentiviral vector).

The effect of siRNA on cell migration was evaluated. Figure 3 indicates that MDA-MB-231 and MDA-MB-468 cell migration is significantly decreased 48 h after siRNA transfection. The relative migration rate of cells transfected with siRNA group was significantly lower compared to the control and NC groups. These results indicate that Col4A2 lentivirus siRNA significantly inhibits the migration of TNBC cells.

### The effect of siRNA on apoptosis of MDA-MB-231 and MDA-MB-468 cells

**Figure 4:**
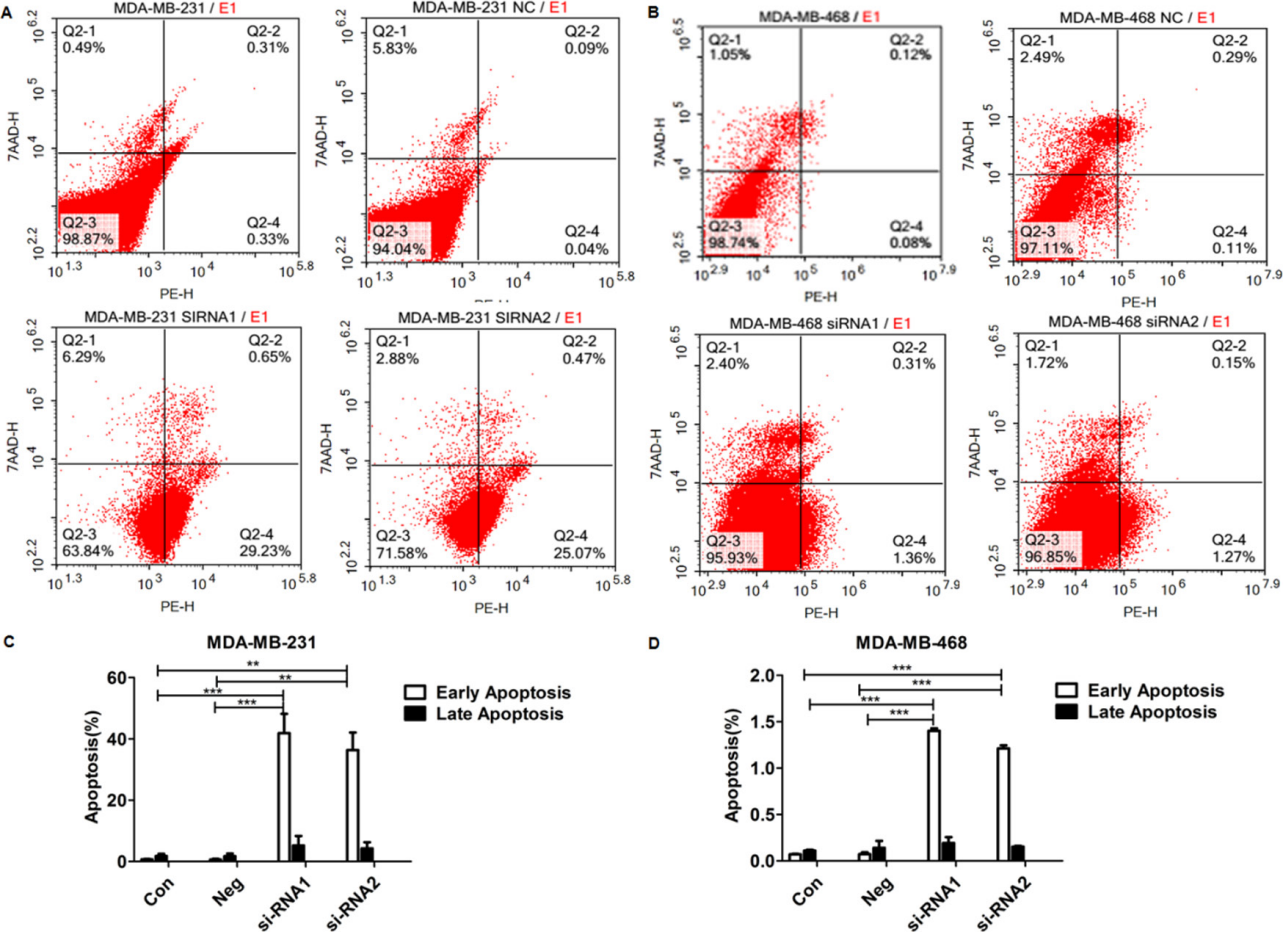
Col4A2 lentivirus siRNA induced apoptosis in TNBC cells FACS results derived from MDA-MB-231 (**A**) and MDA-MB-468 (**B**) in three groups; Early and Late Apoptosis, (%)of MDA-MB-231 (**C**) and MDA-MB-468 (**D**). **P < 0.001; ***P < 0.0001. (Con = non-transfected cells; Neg = cells transfected with control shRNA lentiviral vector).

As shown in Figure 4, in both MDA-MB-231 and MDA-MB-468 cells, after Col4A2 interference treatment, the cells undergoing early apoptosis were significantly increased in the siRNA1 and siRNA2 groups, compared with the NC and control groups. However, siRNA transfection did not significantly affect the number of cells undergoing late apoptosis (Figure 4). These results indicate that the Col4A2 lentivirus siRNA decreased TNBC cell growth by inducing apoptosis.

### Cell cycle of MDA-MB-231 and MDA-MB-468 cells

**Figure 5:**
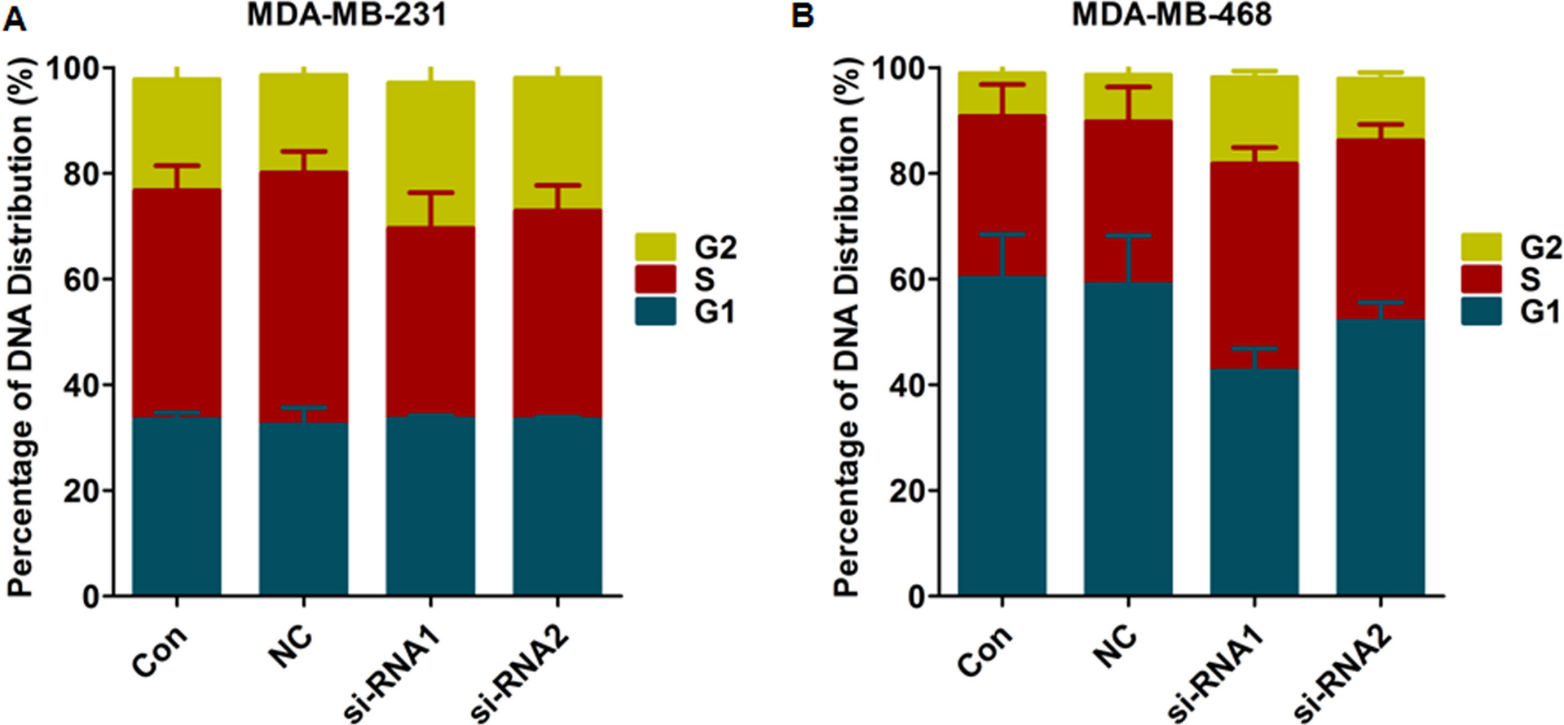
Cell cycle in each group (**A**) MDA-MB-231; (**B**) MDA-MB-468. (Con = non-transfected cells; Neg = cells transfected with control shRNA lentiviral vector).

As shown in Figure 5, after Col4A2 interference transfection, there was no significant difference in the number of cells in the G0/G1 phase compared to the NC and control groups. In contrast, a higher percentage of cells in the G2 phase and a lower percentage of cells in the S phase were detected in the siRNA group compared to the NC and control groups. These results indicate that transfection with Col4A2 lentivirus siRNA arrested the TNBC cell cycle in the G2 phase.

## DISCUSSION

TNBC is a highly invasive clinical subtype of breast cancer characterized by lack of effective targeted therapies, aggressive phenotype and poor prognosis. Therefore, it is important to identify new therapeutic targets for TNBCs. In our previous study, gene expression analysis was performed by microarray to compare gene expression profiling of TNBC to that of non-TNBC. Our previous results demonstrated that Col4A2 was the most significant up-regulated gene in the integrated analysis, and the high expression of Col4A2 in TNBC was further confirmed by qRT-PCR in MDA-MB-231 cells. Protein expression analysis showed that Col4A2 protein was up-regulated. Jak-STAT signaling pathway was found to be highly active for differentially expressed genes including Col4A2 in cancer [27]. It was suggested that the JAK/STAT was one of the TNBC-specific regulatory feedback programs, and its critical signaling nodes could be used as the therapeutic target for the treatment of TNBC [28].

In the present study, we constructed the COL4A2 siRNA lenti-viral vector and transfected it into MDA-MB-231 cells and MDA-MB-468 cells. Col4A2 mRNA and protein expressions were effectively down-regulated in MDA-MB-231 and MDA-MB-468 cells after siRNA lentivirus transfection. The Col4A2 siRNA-transduced cells showed inhibition in cell proliferation and migration in MDA-MB-231 and MDA-MB-468 cells, indicating the migration and proliferation of TNBC cells were regulated by Col4A2 expression. Some classical pathways such as Wnt signaling pathway, PI3K-Akt signaling pathway, Jak-STAT signaling pathway, MAPK signaling pathway, ErbB signaling pathway [29] and some others together may regulate tumor growth and metastasis based on integrated analysis. These results may provide us new insight for the prevention and treatment of TNBC [27]. Apoptosis is an active process of cellular self-destruction, not only functions to limit viral replication by eliminating infected cells but also contributes to limit viral dissemination. After Col4A2 interference treatment in both MDA-MB-231 and MDA-MB-468 cells, the cells undergoing early apoptosis were significantly increased in the siRNA1 and siRNA2 groups, compared to the NC and control groups. However, siRNA did not significantly affect the number of cells undergoing late apoptosis. These results indicate that the Col4A2 lentivirus siRNA decreased the proliferation of TNBC cells by inducing apoptosis. In the future, we might be able to induce programmed cell death through the use of a targeted Col4A2 inhibitor, in order to achieve the goal of treating TNBC. As shown in Figure 5, a higher percentage of cells in the G2 phase and a lower percentage of cells in the S phase were detected in the siRNA group compared to the NC and control groups. These results indicate that transfection with Col4A2 lentivirus siRNA arrested the TNBC cell cycle in the G2 phase to suppress DNA synthesis.

In conclusion, we showed that Col4A2 can restrain both MDA-MB-231 and MDA-MB-468 cells proliferation, migration, cell cycle, and ultimately lead to apoptosis. The data indicated that Col4A2 was significantly correlated with the proliferation and invasive potential of TNBC. Moreover, the result of Col4A2 inhibition can suppressed cell proliferation and invasion in MDA-MB-231 cells and MDA-MB-468 cells which may help to understand the pathogenesis of TNBC. Inhibition of Col4A2 might represent a new target for the prevention and treatment of TNBC. Further *in vivo* and *in vitro* experiments are needed to uncover the biological function of the differentially regulated genes in the pathogenesis of TNBC. A future challenge will be to determine the mechanisms of Col4A2 in regulating breast cancer incidence.

## MATERIALS AND METHODS

### Construction of Col4A2 RNAi lentiviral vector

Specific primers for Col4A2-si-RNA, as well as negative controls (NC), were designed based on the gene sequence in GenBank (Gene ID: 1284), which can be found in Table 1. The pGLV3/H1/GFP+Puro vector was used for selecting clones. All the procedures were performed according to the manufacturer's instructions.

**Table 1:** Primers of Col4A2 si-RNA and negative controls

	Primers(5′–3′)
Si-1 F	GATCCGGGACCCAACGGGATTCCATTTCAAGAGAATGGAATCCCGTTGGGTCCCTTTTTTG
Si-1 R	AATTCAAAAAAGGGACCCAACGGGATTCCATTCTCTTGAAATGGAATCCCGTTGGGTCCCG
Si-2 F	GATCCGGGAATGCAGATGTACAGAATTCAAGAGATTCTGTACATCTGCATTCCCTTTTTTG
Si-2R	AATTCAAAAAAGGGAATGCAGATGTACAGAATCTCTTGAATTCTGTACATCTGCATTCC CG
shNC F	GATCCGTTCTCCGAACGTGTCACGTTTCAAGAGAACGTGACACGTTCGGAGAACTTTTTTG
shNC R	AATTCAAAAAAGTTCTCCGAACGTGTCACGTTCTCTTGAAACGTGACACGTTCGGAGAACG

“F”: Forward; “R”: Reverse.

### Lentivirus packaging and titer measurement

Human embryonic kidney 293T cells (Beijing Dingguo Changsheng Biotech Co., Ltd., Beijing, China) were selected for packaging and titer measurement of the lenti-virus. Cells were cultured in Dulbecco's modified Eagle's medium (DMEM; Gibco, Carlsbad, USA), supplemented with 10% heat-inactivated fetal bovine serum, 100 mg/L streptomycin, and 100 U/mL penicillin (Gibco) under 5% CO2, at 37°C in a humidified incubator. When cells were grown to approximately 70–80% confluence, they were transfected with NC (control shRNA lenti-viral vectors) and Col4A2 RNAi lentiviral vectors using Lipofectamine2000 (Invitrogen, Carlsbad, USA). After 48h, the viruses were harvested and concentrated, followed by titer detection. A serial dilutions of the lentivirus from 10^−1^ to 10^−4^ were prepared by diluting the concentrated lentivirus. 4 days later, fluorescent cells were counted in the last two concentrations, then divided by their corresponding n dilution factors to obtain the original virus titer.

### Transfection

In this study, the effects of down-regulated COL4A2 expression were investigated in two types of human breast cancer cell lines, MDA-MB-231and MDA-MB-468. When cells were grown to approximately 90% confluence, polybrene was added according to the manufacturer's instructions. Then different vector was added according to the MOI ( multiplicity of infection) value and the virus titer, and incubated overnight under 5% CO2, at 37°C. Both cell types were divided into three subgroups: Con-transfected with empty lenti-virus vector, NC group-transfected with NC vector ands iRNA group-transfected with the Col4A2 RNAi lenti-viral vector.

### Real-time PCR

Total RNA was extracted from the cultured MDA-MB-231and MDA-MB-468 cells and the concentration of extracted RNA was estimated by optical density measurement (A260/A280 ratio) with a Q5000 Spectrophotometer (Quawell, Sunnyvale, USA). Real-time PCR amplification was carried out using the SYBR Green-based PCR Master Mix (Applied Biosystems/Life Technologies, Carlsbad, CA). The ABI PRISM 7500 system (ABI, Grand Island, NY, USA) was used for all reactions in a total volume of 25 μL.

### Western blotting

Cells were harvested and total protein was isolated using RIPA buffer (50 mM Tris-HCl at pH 7.4, 1 mM EDTA, 150 mM NaCl, 1% NP-40 and 0.5% SDS) supplemented with proteinase cocktail (Roche, Switzerland), and heated for 5 min at 100°C. Pierce BCA Protein Assay Kit (Thermo, USA) was used to determine protein concentration. Equal amount of denatured protein samples were loaded and separated by 12% SDS-polyacrylamide gels, and then transferred onto polyvinylidene difluoride membranes (PALL). After blocking with 5% non-fat milk power in Tris-buffered saline/0.05% Tween 20 (TBST), the membrane was incubated with a specific primary antibody, followed by the horseradixh peroxidase-conjugated secondary antibody. Proteins were visualized using ECL reagents (Pierce).

### CCK8 assay

After transfection for 72 hours, MDA-MB-231 and MDA-MB-468 cells were incubated for 0, 24, 48, and 72 hours in 96-wells plate with 2000 cells/well. Then 20 μL CCK8 (Dojindo Molecular Technologies, Inc.,Xiongben, Japan) was added to each well for 1 hour, followed by light absorbance measurement at a wavelength of 450 nm.

### Transwell assay

After incubating for 72 hours, MDA-MB-231 and MDA-MB-468 cells were seeded in the upper chambers of 6-well transwell with 25,000 cells/well. The chambers were then incubated for 48 hours at 37°C in a humidified incubator with 5% CO_2_. Subsequently, cells in the lower chamber was stained with hematoxylin and photographed.

### Flow cytometry analyses

For cell cycle analysis, MDA-MB-231 and MDA-MB-468 cells were seeded in the 6-well plates with 500,000 cells/well, and then incubated at 37°C in a humidified incubator with 5% CO_2_. After transfection for 72 hours, the cells were collected, washed with Dulbecco's phosphate buffered saline (DPBS; Genview, El Monte,USA), fixed in 70% ethanol, and incubated overnight at −20°C. Ethanol was removed by centrifugation. The cell pellets were washed with DPBS, followed by incubation with 100 μL propidium iodide (PI, Sigma, St.Louis, USA) solution for 5–10 minutes in the dark at 37°C. The cells were analyzed using a flow cytometer (Beckman Coulter, Brea, USA).

For apoptosis analysis, cells were stained using the Annexin V-FITC Apoptosis Detection Kit (ShanghaiGenechem Biotech Co., Ltd.) and PI staining. Cells were harvested 72 hours after transfection, binding buffer (5 μL Annexin V/FITC) was added according to the manufacturer's instructions. After 15 minutes, PI staining was performed in the dark at room temperature, followed by incubation in the dark at room temperature for another 15 minutes. Apoptosis was then detected by flow cytometry.

### Statistical analysis

Data are shown as the means ± standard deviation. The statistical significance of differences between groups was assessed via one-way analysis followed by Student's *t*-tests of comparison. *p*-values less than 0.05 were considered to be statistically significant. Statistical analyses were conducted using the GraphPad Prism 4.0 software.
